# 18.2 USING COMPUTATIONAL ESTIMATES OF INTERNAL “NOISE” TO CHARACTERIZE VISUAL PERCEPTUAL AND WORKING MEMORY DEFICITS IN SCHIZOPHRENIA

**DOI:** 10.1093/schbul/sby014.071

**Published:** 2018-04-01

**Authors:** Megan Ichinose, Woon Ju Park, Duje Radin, Sohee Park

**Affiliations:** 1 Vanderbilt University; 2 University of Rochester

## Abstract

**Background:**

Heightened neural noise serves as a promising explanatory framework for schizophrenia (SZ) pathophysiology, yet its specific contribution to working memory (WM) deficits remains unclear. The perceptual template model (PTM), an established human-observer model of visual perception, asserts that a system’s internal noise (IN) is due to both background, ‘additive’ noise and stimulus-driven ‘unfiltered’ noise. In this study, we assessed levels of PTM-derived additive and unfiltered IN in SZ during basic visual processing and tested their respective relations to patients’ visuospatial WM imprecision.

**Methods:**

Individuals with SZ and demographically-matched healthy controls completed a perceptual discrimination task to estimate levels of IN and an analog visual WM task to examine the impact of internal noise on WM precision. The discrimination task involved distinguishing orientations of briefly presented gratings (1 cycle/°; tilted ±45° from vertical) embedded in varying levels of external noise (0–21%). Contrast thresholds were estimated, and additive and unfiltered IN levels were modeled from task performance with the PTM. The WM task required reproducing remembered orientations of high-contrast gratings (same size and spatial frequency as in the discrimination task) with a manual dial at a 1s delay. WM precision was computed as the concentration of the von Mises distribution, fit from subjects’ orientation errors.

**Results:**

Additive and unfiltered IN during perceptual discrimination were both significantly increased in SZ compared to HC. WM precision was reduced in SZ compared to HC at every set size. Levels of unfiltered IN negatively correlated with WM precision in SZ, while both unfiltered and additive IN negatively correlated with WM precision in HC. For SZ, unfiltered IN was also negatively correlated with IQ, and WM precision was positively correlated with IQ in both groups.

**Discussion:**

We found evidence of elevated IN levels during visual perception in SZ, though only unfiltered IN was inversely related to patients’ visual WM precision. Thus results indicate overall ‘noisy’ visual perception in SZ, but point to a more precise model of poorer signal filtering or noise suppression as contributing to WM deficits and potentially broader cognitive impairment. Future work must identify the neural drivers of IN levels, as they may shed light on differential implications of the excitation/inhibition imbalance in WM networks. Findings underscore the link between perception and WM encoding in SZ and offer a novel computational strategy for identifying common and unique pathophysiological mechanisms of SZ cognitive dysfunction.

